# Nationwide Industry-Led Community Exercise Program for Men With Locally Advanced, Relapsed, or Metastatic Prostate Cancer on Androgen-Deprivation Therapy

**DOI:** 10.1200/OP.21.00745

**Published:** 2022-05-18

**Authors:** Oliver Schumacher, Daniel A. Galvão, Dennis R. Taaffe, Nigel Spry, Dickon Hayne, Colin Tang, Raphael Chee, Robert U. Newton

**Affiliations:** ^1^ Exercise Medicine Research Institute, Edith Cowan University, Joondalup, WA, Australia; ^2^ School of Medical and Health Sciences, Edith Cowan University, Joondalup, WA, Australia; ^3^ Medical School, Surgery, University of Western Australia, Perth, WA, Australia; ^4^ Urology Department, Fiona Stanley Hospital, Murdoch, WA, Australia; ^5^ Department of Radiation Oncology, Sir Charles Gairdner Hospital, Nedlands, WA, Australia; ^6^ GenesisCare, Joondalup, WA, Australia

## Abstract

Androgen-deprivation therapy in patients with prostate cancer (PCa) is associated with considerable side effects and secondary comorbidities such as overweight/obesity and cardiovascular disease. The aim of this study was to investigate the effectiveness of an industry-led, treatment-integrated, community-based exercise program on outcomes of body weight, cardiovascular health, and physical function.

**PATIENTS AND METHODS:**

PCa patients with locally advanced, relapsed, or metastatic disease receiving leuprorelin acetate were enrolled across multiple sites in Australia and assigned supervised group exercise undertaken weekly or biweekly (ie, 16 exercise sessions in total) for 10-18 weeks, consisting of aerobic and resistance training performed at moderate-to-vigorous intensity.

**RESULTS:**

Between 2014 and 2020, 760 participants completed the baseline and follow-up assessment. Participants were age 48-94 years, and most were either overweight (42.1%) or obese (38.1%). Program compliance was high, with 90% of participants completing all 16 exercise sessions. There was a small but significant reduction in waist circumference (–0.9 cm; 95% CI [–1.2 to –0.5]; *P* < .001) and no change in weight or body mass index. Systolic (–3.7 mmHg; 95% CI [–4.8 to –2.6]; *P* < .001) and diastolic (–1.7 mmHg; 95% CI [–2.3 to –1.0]; *P* < .001) blood pressure were significantly lower after the program. Furthermore, significant improvements were seen in cardiorespiratory fitness and muscle strength (*P* < .001). For most of the investigated outcomes, participants with poorer initial measures had the greatest benefit from participating in the program.

**CONCLUSION:**

The community exercise program was feasible and effective in preventing weight gain, reducing blood pressure, and improving physical function in patients with PCa on androgen-deprivation therapy.

## INTRODUCTION

Androgen-deprivation therapy (ADT) is an effective and extensively used treatment option in the management of locally advanced and metastatic prostate cancer to delay disease progression.^1-3^ However, ADT is associated with a number of adverse effects including hot flushes, loss of libido, lethargy, decreased bone mineral density, and reduced lean mass, as well as increased fat mass.^4-7^ Overweight and obesity are a common treatment-related adverse effect of ADT, and postdiagnosis weight gain has been associated with increased all-cause mortality and poorer prostate-specific outcomes.^8^ Moreover, testosterone suppression has been linked to increased risk of developing cardiovascular disease.^9,10^ As a result, comorbidities such as overweight/obesity and cardiovascular disease are prevalent in men with prostate cancer and may negatively affect physical function, disease progression, and quality of life.^11-13^

Exercise has been identified as an effective strategy to counter or mitigate many of these treatment-related toxicities in well-controlled research settings.^14,15^ However, addressing the adverse effects of ADT on fat mass has proven difficult in men with prostate cancer, and the impact of self-managed long-term exercise uptake is largely unknown in this patient population.^16,17^ Furthermore, widespread adoption and implementation of exercise programs into clinical care pathways is scarce and thus, access to appropriate exercise programs for patients outside of research trials is limited.^18^

The Man Plan program is an ongoing Australia-wide industry-led community-based exercise and support program for men diagnosed with locally advanced, relapsed, or metastatic prostate cancer treated with leuprorelin acetate (Lucrin, AbbVie Pty Ltd, Mascot, NSW, Australia), and is considered an integrated part of treatment for these patients.^19^ The primary aim of this study was to evaluate the effectiveness of this program and investigate the impact on body weight, cardiovascular health, and physical function outcomes in a large cohort of patients with prostate cancer treated with ADT. The secondary purpose of this study was to investigate whether demographic and treatment characteristics moderate the effects of the exercise program.

## PATIENTS AND METHODS

### Study Design

The Man Plan is a support program for men with prostate cancer undergoing treatment with leuprorelin acetate and includes exercise with three forms of delivery: (1) supervised group exercise, (2) home-based exercise, and (3) a support program for those patients unable to exercise. Prostate cancer patients with locally advanced, relapsed, or metastatic disease receiving leuprorelin acetate were enrolled into the program by invitation from their attending specialist across different sites in Australia. Group allocation is based on patient preference (in consultation with their health care provider), presence of comorbidities, and individual fitness levels. Patients were excluded from the supervised and home-based exercise programs and allocated to the support only program (ie, no formal exercise) if they had any musculoskeletal, cardiovascular, or neurologic disorders that prevented them from exercising, were unable to walk 400 m, or could not perform upper and lower limb exercises (eg, because of symptomatic bone metastases or extensive metastatic disease). Medical consent was provided at referral to the exercise program via a dedicated website.^20^ The study was approved by the Human Research Ethics Committee at Edith Cowan University, and all patients provided written informed consent before participation. The present analysis of the Man Plan focuses exclusively on outcomes of participants in the supervised exercise program.

### Exercise Program

The community-based exercise program consisted of one to two exercise sessions each week supervised by an accredited exercise physiologist (AEP) and was conducted over a total of 10-18 weeks, depending on participant attendance (16 exercise sessions in total plus initial and final assessment). The exercise prescription was modeled after a previously conducted exercise trial in men with prostate cancer receiving ADT.^21^ In brief, progressive resistance training was performed at 6- to 12-repetition maximum and two-four sets per exercise for upper-body and lower-body muscle groups. Resistance training was supplemented with up to 20 minutes of aerobic exercise at 65%-80% of maximum heart rate or a perceived exertion of 11-13 (6-to-20-point Borg scale). However, the exercise program was tailored to each patient's needs on the basis of professional judgment of their assigned AEP.

### Outcome Measures

For participants in the supervised exercise program, height, weight, waist circumference, cardiovascular variables (systolic and diastolic blood pressure, and resting heart rate), aerobic capacity, and muscle strength were assessed at baseline and after the final exercise session of the program. Aerobic capacity (cardiovascular fitness) was assessed using the 400-m walk test.^22^ The strength of upper-body and lower-body muscle groups was assessed with the leg press, chest press, and seated row exercises. Specifically, the weight that could be lifted only 10 times was recorded for each exercise (ie, 10-repetition maximum testing). In addition, the number of modified (ie, knee) push-ups that could be completed in 30 seconds was recorded. All measurements were collected by an AEP. Where participants completed the supervised exercise program more than once, only assessments corresponding to the first time the program was completed were included in the analysis. Program satisfaction, subjective effectiveness of the exercise intervention on fitness levels, and attitudes toward exercise were assessed at the conclusion of the exercise program; surveys were conducted via telephone by a program coordinator.

### Statistical Analysis

Data were analyzed using R version 4.1.0 (The R Foundation). For continuous variables, normal distribution of the difference between preintervention and postintervention scores was assessed visually using Q-Q plots. Only data from participants who completed both the preintervention and postintervention assessments were included in the analysis. Descriptive data are presented as mean ± standard deviation, mean difference (MD; 95% CI), or n (%), unless indicated otherwise. Variables measured at baseline (pre) and after 10-18 weeks (post) were compared using paired *t*-tests. Effect sizes are based on Cohen's *d* with the root mean square of preintervention and postintervention standard deviation as the denominator.^23^ Multiple linear regression was used to investigate the association between participant characteristics (independent variables) and change in outcome measure (dependent variable). Individual regression analyses were performed for each outcome separately with age, time since diagnosis, time since first Lucrin injection, Lucrin injection frequency, family history of prostate cancer, program compliance, and the baseline value of the outcome included as independent variables in the model. All statistical tests were two-tailed, and *P* < .05 was considered statistically significant.

## RESULTS

From 2014 to 2020, 1,515 participants referred to the Man Plan program were allocated to the supervised exercise group at participating sites across Australia. Of these, 760 participants (50%) completed anthropometric, cardiovascular, and exercise performance assessments at baseline as well as after the supervised exercise program and are reported upon in this analysis. The mean age of participants was 72 ± 7 years and ranged from 48 to 94 years. The majority of participants were either overweight (42.1%) or obese (38.1%); and 128 and 32 men, respectively, reported having an immediate or extended family history of prostate cancer. Median time between receiving their first Lucrin dose and baseline assessment was 0.85 months, and quarterly injections (ie, every 3 months) was the most common treatment schedule. Importantly, the participants who did not complete the follow-up assessment were not substantially different in terms of their baseline characteristics from those participants who did complete the follow-up assessment after the supervised exercise program, although median time between receiving their first Lucrin dose and baseline assessment was slightly longer at 0.98 months (*v* 0.85 months; *P* = .032).

### Program Compliance

Exercise session attendance data were available for 731 participants (96%) who completed both the initial and final assessments. Overall, 98% of the planned exercise sessions were completed for these participants. Six hundred fifty-nine participants (90%) completed all 16 allocated exercise sessions and only four participants attended < 50% of the program. Considering only participants who completed the initial but not the final assessment, 53% of the total planned exercise sessions were completed (on the basis of exercise session attendance data available for 619 participants [82%] of the participants who did not complete the final assessment). Of these, 103 participants (17%) still completed all 16 allocated exercise sessions but did not attend the final assessment. Furthermore, the median number of sessions attended by the participants who did not complete the final assessment was eight, and 295 participants (48%) attended < 50% of the program.

### Anthropometric Measures

Anthropometric measures at baseline and postintervention are presented in Table 1. Following the exercise program, there were no significant changes in either weight (MD = –0.1 kg; 95% CI [–0.3 to 0.1]; *P* = .331) or body mass index (BMI; MD = –0.03 kg/m^2^; 95% CI [–0.09 to 0.03]; *P* = .327); however, there was a significant, albeit modest, reduction in waist circumference (MD = –0.9 cm; 95% CI [–1.2 to –0.5]; *P* < .001). Changes in anthropometric measures were significantly associated with baseline values of the outcome, suggesting that participants with higher initial values had a greater benefit from the program (Table 2 and Data Supplement, online only). Furthermore, better program compliance was significantly associated with a greater reduction in waist circumference.

**TABLE 1.**
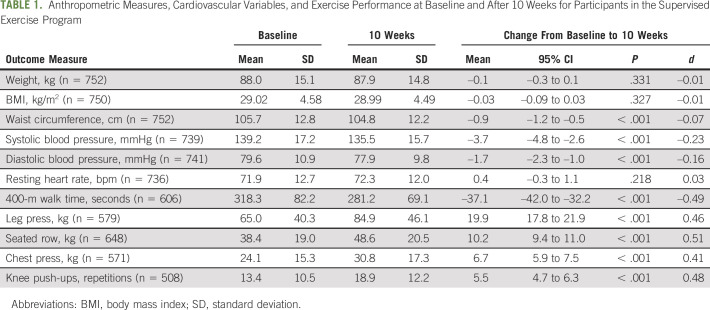
Anthropometric Measures, Cardiovascular Variables, and Exercise Performance at Baseline and After 10 Weeks for Participants in the Supervised Exercise Program

**TABLE 2.**
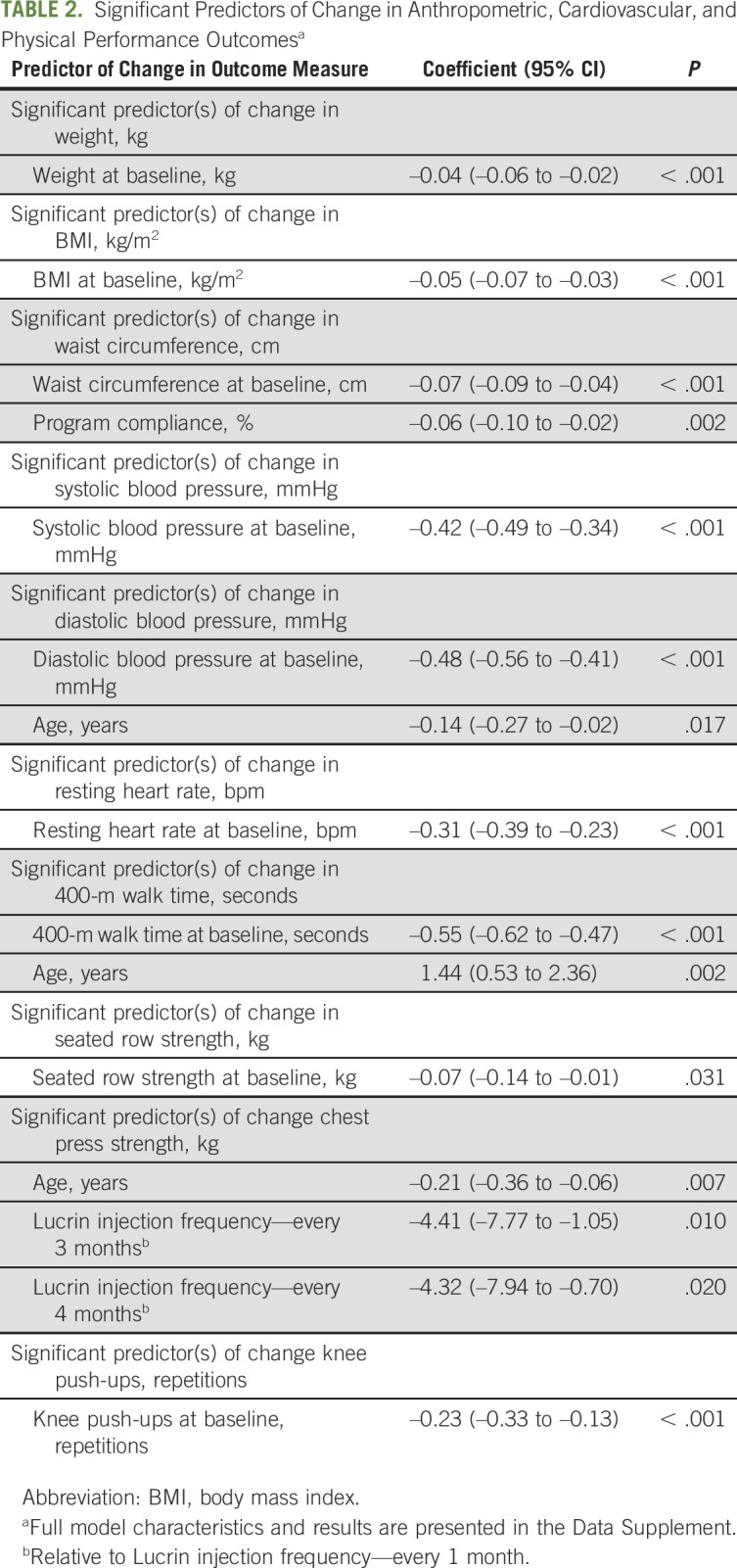
Significant Predictors of Change in Anthropometric, Cardiovascular, and Physical Performance Outcomes^a^

### Cardiovascular Health

Cardiovascular variables of systolic blood pressure, diastolic blood pressure, and resting heart rate before and after the exercise intervention are presented in Table 1. There was no significant change in resting heart rate (MD = 0.4 bpm; 95% CI [–0.3 to 1.1]; *P* = .218); however, both systolic and diastolic blood pressure were reduced by –3.7 mmHg (95% CI [–4.8 to –2.6]; *P* < .001) and –1.7 mmHg (95% CI [–2.3 to –1.0]; *P* < .001), respectively, following the exercise intervention, constituting small-to-negligible effects. As with anthropometric measures, systolic and diastolic blood pressure as well as resting heart rate reductions were significantly greater in participants with higher baseline values (Table 2 and Data Supplement). In addition, participants showed a significantly greater reduction in diastolic blood pressure with increasing age.

### Cardiorespiratory Fitness and Neuromuscular Strength

Physical function outcomes are presented in Table 1. The exercise intervention resulted in significant improvements (*P* < .001) across all outcome measures, with effect sizes ranging from small (*d* = 0.41) to medium (*d* = 0.51). Participants with slower initial 400-m walk times showed significantly greater performance improvements (Table 2 and Data Supplement). However, older age was significantly associated with larger decrements in 400-m walk performance. Similar findings regarding the impact of age were observed for the chest press exercise, although the effect was minimal. There were no significant moderators of leg press strength.

### Program Satisfaction

Among participants who completed the survey following the supervised exercise program, 99% indicated they enjoyed the exercise sessions and 97% reported they felt that the exercise sessions helped increase their level of fitness (Table 3). Furthermore, being part of an exercise group motivated 92% of participants to keep going back to the exercise sessions and 97% felt that the Man Plan program had improved their overall well-being. The majority of participants (94%) indicated that they planned to continue exercising after the program, with approximately 40% stating that they would keep in touch with their exercise physiologist and/or exercise at home, and 24% intending to join a fitness facility.

**TABLE 3.**
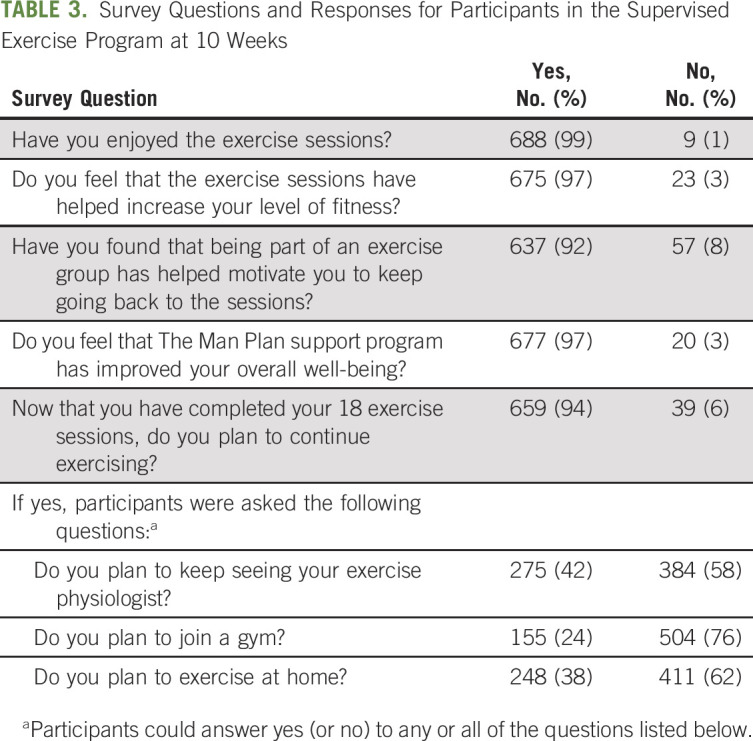
Survey Questions and Responses for Participants in the Supervised Exercise Program at 10 Weeks

## DISCUSSION

The Man Plan program is an Australia-wide, industry-led, treatment-integrated, exercise and support program for men with prostate cancer receiving leuprorelin acetate. In this study, we report the findings before and after the supervised, community-based exercise program and the effects on body weight, cardiovascular health, and physical function, as well as self-reported outcomes of program satisfaction, subjective effectiveness, and attitudes toward exercise. The exercise sessions were well attended, and the program was overwhelmingly well received by participants who noted that exercise was beneficial for improving their level of fitness and overall well-being. These findings are substantiated by objectively measured outcomes; the program resulted in a significant reduction of blood pressure, was successful in maintaining body weight, and improved components of physical function, including cardiorespiratory fitness as well as upper-body and lower-body muscle strength. Importantly, those with poorer initial outcomes showed greater improvements from participating in the exercise program.

A common adverse effect of ADT is obesity-related comorbidity such as cardiovascular disease and diabetes.^24^ Although the underlying biological mechanisms of this link are not fully understood, the widespread use and clinical benefit of ADT require strategies to be developed to counteract these common side effects of treatment. Studies have indicated that structured exercise may represent a promising approach in this regard.^25^ Here, we showed that the Man Plan program was successful in maintaining body weight, which can be seen as a positive outcome, given the expected weight gain and detriments in body composition associated with ADT. Conversely, given the BMI category of the patient population in this study (ie, overweight [25-29.9 kg/m^2^]), waist circumference values remained elevated (ie, ≥ 100 cm) after the program, despite a slight reduction.^26,27^ Indeed, BMI has been found to not significantly change after exercise interventions in men with prostate cancer, presumably as a result of concomitant reductions in fat mass in combination with gains in lean mass.^28^ However, our analysis lacks a control group with nonexercise participants for comparison, and further quantification of a potential beneficial shift in lean mass as well as fat mass was not possible, thus preventing more specific conclusions regarding body composition.

In a previous analysis, Taaffe et al^29^ found that most men receiving ADT responded favorably to a resistance-based multimodal exercise program in terms of benefits for body composition, muscle strength, and physical function. These findings are supported by this study, with almost all participants improving in at least one of the assessed outcome measures (individual data not shown). Furthermore, the changes in muscle strength and physical function as well as systolic and diastolic blood pressure are consistent with the results of randomized controlled trials, with changes in physical function being slightly larger in this study, whereas changes in systolic and diastolic blood pressure were slightly smaller compared with randomized controlled trial results.^30,31^ Unsurprisingly, participants with poorer baseline values had a better response to the exercise program. This result is in line with previous data, where it was shown that, for example, fatigue and psychologic distress were alleviated the most in men with prostate cancer who had the highest symptom burden at baseline.^32,33^ Hence, if well enough to exercise (ie, presenting with no imminent contraindications), patients with initially high symptomatology that would perhaps not be considered suitable for exercise should be considered for referral to a qualified exercise professional after careful evaluation as they may represent the population to gain the most from participating in a structured and supervised exercise program.

The Man Plan program was well received by participants who provided feedback at the end of the intervention, and they felt that the exercise sessions had helped improve their fitness and overall well-being. Furthermore, the group setting was motivating to maintain a regular exercise regimen as stated by the men. In line with the results of this study, Reale et al^34^ reported that all participants in a supervised exercise program for men on ADT embedded into standard prostate cancer care believed that exercise has beneficial effects on physical, psychologic, and social aspects. They also pointed out that it is possible to deliver national care recommendations on exercise by working together with community partners.^34^ Similarly, the Man Plan was initiated by industry sponsors in collaboration with public research institutions and delivered by existing services in the community across Australia. These partnerships and networks are crucial to provide sustainable, comprehensive, and widespread care to patients with prostate cancer during treatment.

A limitation of this analysis is the entirely observational nature of this cohort study with a group of patients with prostate cancer receiving exercise and hormone therapy as part of their cancer management but no comparator group of patients receiving hormone therapy and no exercise. However, we have illustrated that the findings from the present analysis are comparable with those of randomized controlled trials in this setting. Moreover, given the exclusion criteria, it is not expected that the participants in the Man Plan, specifically the supervised exercise group, would substantially differ from those of other trials in this patient population. A further potential limitation of this study is the patient-dictated intervention allocation process of the Man Plan program, which may have resulted in self-selection as well as positive outcome bias that could have led to favorable findings owing to participants being more motivated to participate in a structured exercise program. However, patient preferences in the intervention allocation process may have helped to increase compliance with the program, thus resulting in superior outcomes that are arguably more reflective of a real-world setting in which patients wish to undertake supervised exercise. Another caveat of this study concerns the linear regression analysis. It should be noted that some of the models explained little of the observed variance, indicating that other variables not assessed in the present investigation are more influential in predicting changes in body weight and waist circumference, blood pressure, and physical function outcomes. Given the scale of the Man Plan program, it was not feasible to collect a more comprehensive set of patient, cancer, and treatment characteristics, but with increasing ease of use of online technologies, extended (eg, online capture of patient-reported outcomes that are directly linked to the study database) and automated (eg, electronic/mobile health devices to assess vital signs and training parameters such as intensity and duration) data-capturing efforts should be considered to better characterize ongoing and future large-scale implementation projects.

In conclusion, The Man Plan program—a treatment-integrated, community-based, and industry-led exercise and support program for men with prostate cancer receiving leuprorelin acetate—is a feasible, effective, and enjoyable intervention to prevent weight gain, reduce blood pressure, and improve physical function. Moreover, patients presenting with the poorest outcome measures at baseline benefited the most from participating in the structured and supervised exercise sessions. Consequently, where possible, exercise as an adjunct therapy for patients with prostate cancer undergoing treatment should be offered to enhance function and well-being.
